# The Combined Appeal for London Hospitals

**Published:** 1922-08

**Authors:** 


### THE COMBINED APPEAL FOR LONDON HOSPITALS.

FOLLOWING the "intensive fortnight" (June 25—July 8) of the Combined Appeal, a dinner was given at the Guildhall by the Lord Mayor and the Council of King Edward's Hospital Fund for London. The Duke of York, taking the place of the Prince of Wales, the President of the Fund, said that nothing short of the very strict orders which his brother had received to rest for the present from public duties would have interfered with his presence on that occasion. The task before the committee was to raise half a million pounds, a large sum, yet small when measured against the mass of human suffering with which the hospitals had to cope. At the zenith of their medical efficiency and soundness of administration, the voluntary hospitals in this country had been brought to the brink of ruin by circumstances quite beyond their own control. Beds had been closed, important branches of healing discontinued, and many schemes of development abandoned. By a great and exceptional effort of public benevolence made now, the vitality of this cherished national organisation would resume its normal state, and the healthy activity of an overstrained system would be restored to the splendour of its former purpose.

Sir Alan G. Anderson, chairman of the Organising Committee, said that £500,000 was a small figure if balanced against the immense sum that the nation would have to find if the voluntary hospital system broke down, and the rates were burdened with the £3,500,000 per annum which these hospitals cost. This appeal, if met, would not by itself put the hospitals permanently on their feet, but it would give them time to turn round. Lord Cave had found that with two years' grace the hospitals could put themselves right by an appeal to a wider public. It had been calculated that 95 per cent. of the charity that flowed to hospitals came from 5 per cent. of the population. That division of the burden would not have been absurd in the Middle Ages when the hospitals started, but it was wrong to-day. We were no longer a nation of rich and poor, but were delicately graded right up and right down, and a large number of those who went to hospitals for their treatment had won the right to call themselves independent men and women. They valued that right, and if the case was fairly put to them he had not the smallest doubt that they would show themselves ready to pay the full cost of their maintenance while in hospital. The public were asked to aid the hospitals in a crisis which was the result of the change from the age of dependence, the Middle Ages, to this age of freedom and independence. Charity was the daily task of the doctor, and because throughout his training he had been taught to give charity to those who came to him, so he valued that side of the work of the hospitals more than many business men would value it. They must not disregard that view. The charity side of the hospitals must continue.

Viscount Cave said our hospital system stood by itself: there was nothing quite like it in the world. There was no place where disease and accident were met to the same extent as they were here by the public spirit of the nation, acting on its own initiative through private direction and with private resources. Hospital work, if national or municipal, tended to become mechanical, and sometimes even perfunctory. But the voluntary hospitals were equipped with the energy and devotion of the foremost physicians and surgeons in the world. They were humanised by the tender care of the nursing staffs, and inspired by the living and personal sympathy of governors and voluntary helpers alike. In the London area there were 120 voluntary hospitals, which provided something like 13,000 beds, and controlled an expenditure of more than £2,500,000 a year. They were not only places for healing: they were places of consultation. Our public infirmaries had no outpatients' departments, and he believed that the attendance on outpatients in the London area alone exceeded 5,000,000 a year. Further, the hospitals had an educationa function, for doctors and nurses were trained in them. He did not doubt that the effect of this appeal and of the Government grants would be to set them on their feet. But we could not have a combined appeal and a Government grant every year, and we must look for some other means of support. He was sure that the stream of private generosity would continue to flow. But we must go a little further. He was a believer in the proposal which had been made for asking all wage-earners to make a regular, though small, contribution, out of their weekly wages, and in the proposal for asking all industrial firms to set side a small percentage of their profits for the same purpose.

Mr. J. H. Thomas, M.P., believed that those who could afford to pay should have the opportunity to pay, but the larger number who could not afford to pay must not be forgotten. This appeal was a real S.O.S. signal. " No man in this audience could dare to picture our great City of London," he said, " if our hospitals went."

Viscount Knutsford said that a hospital chairman had to combine the manners of a gentleman with the instincts of a felon. He inherited the first and caught the second from his fellow-chairmen. He hoped the public would realise that this appeal was made to get the hospitals out of difficulties into which they had drifted. The hospitals must not be left so much to the chance of an appeal, or a legacy, or of some rich man thinking he must buy salvation. They had existed on chance too long. If they could not go on, the only alternative was to become State hospitals, and the thought of that saddened him.

Mr. E. S. Shrapnell-Smith, Director-General of the Appeal, said the total that night was £253,000, an increase of £26,000 since noon. He was pleased to acknowledge receipt of a personal cheque that evening from the Prince of Wales for 100 guineas. The Lord Mayor's Fund now totalled £56,403. The sum of £49,201 was collected on Empire Flag Day, while the Albert Hall Ball brought in £8,000. The leading London boroughs were Hampstead (£6,279) and Ealing (£3,250).

In connection with the scheme by which the schools of Greater London intend to help the Combined Appeal, a conference of headmasters and headmistresses in London beyond the jurisdiction of the L.C.C. was held on July 10 at King's College. Prof. Winifred Cullis appealed to the teachers because nobody in the world could and would work harder for ideals. The amount it was hoped the schools would raise was £50,000, and they appealed to art schools, dramatic schools, commercial and medical schools, as well as the ordinary educational schools. Lord Burnham believed that the Schools' Hospital Fund was the hope and strength not only of the Combined Appeal, but of the hospital movement. The reason why the main appeal had fallen short up to the present of what was requisite—he did not say it would ultimately fall short—was that in this great quarter of the country they had not the hospital habit of mind. In the provincial cities the hospital habit had been carried so far that in many cases a third of the whole population were subscribers to the local hospitals, and they practically provided the whole of the money necessary for the upkeep and general development of hospital equipment. The citizens of London were not one whit less patriotic or less responsive, but they were ignorant of the necessities of the case. Therefore, when schools of all kinds and classes undertook this great work they were going to do far more than collect money ; they were going to create the hospital conscience.

The Executive Committee of the Appeal have recommended the Council of King Edward's Hospital Fund to make an early distribution of at least £200,000 from the money already received. As we went to press the gross total proceeds from all sources amounted to £273,700.

